# 

**Published:** 1887-07-23

**Authors:** 


### The Hospital.

**An Institution, Family, and Parochial Journal of Hospitals, Asylums, and all Agencies for the Care of the Sick, Criticism and News.**

SATURDAY, JULY 23RD, 1887.

### NOTICE TO CORRESPONDENTS.

All correspondence must be written on one side of the paper only.
We cannot undertake to return MSS. not used.

**Communications**, whether intended for insertion or for private information, must be authenticated by the names and addresses of the writers, not necessarily for publication.

**Local papers** containing reports or news paragraphs **should be marked.**

It is especially requested that early intelligence of **local events of interest**, or which it may be thought desirable to bring under notice, may be sent direct to the Editor.

Communications respecting editorial matters should be addressed to the Editor, Norfolk House, Norfolk Street, Strand, London, W.C.

### NOTICE TO ADVERTISERS.

*To insure prompt insertion, all Advertisements for* THE HOSPITAL *must be sent in direct to A. P. Watt, 2, Paternoster Square, E.C.*

### THE LITERARY PRIZE.

A PRIZE of **Two Guineas** will be given every month for the best story or incident based upon hospital, or asylum, or student life, or any incident connected with the professions of medicine or nursing.

### The Children's Corner.

**Fourth Quarterly Prize Competition.**
Began 2nd July, 1887 : ends 24th Sept., 1887.
PRIZES.
FOR MOST CORRECT ANSWERS TO PUZZLES.

| | | | |
|---|---|---|---|
| 1st .. .. | Thirty Shillings | 3rd .. .. | Ten Shillings |
| 2nd .. .. | Twenty " | 4th .. .. | Five " |

FOR NEATEST ANSWERS TO PUZZLES.
Five Shillings.
FOR BEST LITTLE LETTERS.
Ten Shillings.

**Puzzles—Fourth Week.**

No. 13. ORNITHOLOGICAL CHARADE.

My *first* is one of royal rank,
Tho' not from trouble free ;
My *next* doth often find a grave
Beneath the treacherous sea ;
My *whole* a shy and timid bird,
Go seek where sedge and rushes wave.

No. 14. CONUNDRUM. What do the sun and moon do to ice ?

No. 15. DIAMOND PUZZLE. 1. A consonant. 2. What all should shun. 3. Snug. 4. Members of a community. 5. To deceive. 6. Anger. 7. The beginning of silence.

No. 16. ANAGRAMS.—RIVERS IN EUROPE. 1. Heron. 2. No anger. 3. May dew. 4. One by.

**Letters—Fourth Week.**

Who can write me the best short story of a *Pic-nic*, in which there might perhaps be a cow, wild or otherwise; a red-currant tart, and a gipsy ? I am sure all of you will have something amusing to tell me on this subject.

**Results of Second Week—Puzzles.**

No. 5. BIBLICAL CHARADE. Aar-on = Aaron.

No. 6. DECAPITATION. All my little friends have guessed Stone, Tone, Ton.

No. 7. SQUARE WORD. The *lights* here are

D U K E
U N I T
K I L N
E T N A

No. 8. FLORAL CHARADE. I have received various answers to this. The correct one is Cows-lip = Cowslip.

*All right*—A. G. S. McCulloch, Mount Edgcumbe, Le Cerf Agile, Mikado, Umslopogaas, Princess Ida, Skye, Ossian, Edith, Poodle, A. C. K., Scrooge, Joe, Alsie ; *Three right*—Toby, Ellie, The Eda ; *Two right*—Violin, Socrates.

**Letters.**

WILDFLOWERS.

I am very much pleased with the letters I have received on this subject. They show a love and appreciation of these lovely objects of Nature, which all true children should possess. The poet Cowper's beautiful lines may be welcome to those who are not acquainted with them.

Nature is but a name for an effect,
Whose cause is God. Not a flower
But shows some touch, in freckle, streak, or stain,
Of His unrivalled pencil. He inspires
Their balmy odours, and imparts their hues,
And bathes their eyes with nectar, and includes,
In grains as countless as the sea-side sands,
The forms with which He sprinkles all the earth."

Mount Edgcumbe, aged 14½, writes a really poetical letter:—

Wildflowers are free to all ; they are the flowers of the poor, childhood's first love, and if their colours are less brilliant or their perfume less strong than those the cultivated garden beauties boast, they are far more graceful ; and what is more delicious than that nameless scent, "the balmy breath of summer fields" ? They are all beautiful, from the pure snowdrop, white as the snow upon whose breast it reposes ; "the daffodil that comes with spring and takes the winds of March with beauty" ; the "violets, sweet violets" beloved by many an humble flower-seller, to the trailing clematis, the old man's beard that seems to cover the bushes with early snow, and more than all, that "little flower with silver crown and golden eye" that is with us all the year, and of which we are told, "The rose has but a summer reign, the daisy never dies." Flora has indeed many devotees. Who would not fly from the rush and turmoil of the world, "far from the busy haunts of men," to rest amidst trees and waving grasses, and loveliest of all wildflowers ?

In this letter, Skye, aged 14, gives an interesting account of some of the wildflowers to be found in Cumberland :—

Since I can remember anything, I was fonder of gathering wildflowers than any other amusement. My favourites among them are honeysuckle and wild roses, and blue cornflower. I found a great quantity of wildflowers in Scotland and Cumberland. We found a very funny berry in Cumberland, called the cloud berry, with a delicate white flower, something like the grass of Parnassus. It grew in the moors, and the berry was something like a raspberry, only hard, and of a lovely scarlet. I think they were called cloud berries, because they grew so high up near the clouds, three thousand feet above the sea. We found any amount of the grass of Parnassus, and the globe buttercup, which I never can find here. I went to a flower-show yesterday, and there were three prizes for wildflower bouquets. They were very nice, but not much variety in them. I should like to learn botany very much, but the worst of people who have just begun it is, they are always airing their grand Latin names of the flowers. I think water-lilies look lovely floating on the water like fairy boats, but when you take them in your hand, or wherever you put them, they look uncomfortable. I can never find any near here. I must not make my letter any longer.

Daisy, aged 10, writes from Ardrossan, N.B. :—

I do not believe I have any particular wildflowers for favourites, they are all so pretty. We have lots round about here. At Port-in-Cross, not far from us, there are the most lovely wildflowers. There are honeysuckle, wild roses, heather, foxgloves, meadow sweet, gowans, yellow and white bedstraw, beautiful vetches, and red and purple gooseberry pie, and many, many more. There is a beautiful white and mauve vetch, so large that it looks something like westeria, mamma says. When we came here, the hedges were white with may, and there were a great many sand roses. The sand roses grow very low, and are pale yellow, with such a sweet smell. They grow for a mile down the coast road, and the air is sweet with their scent. On a sunny day they look nearly white, and the sea so sparkling and blue, and Arran so beautiful with even its fields to be seen ; and Ailsa Craig in the distance looking a misty blue. The view is so lovely, and the roses so sweet, that I almost think they are very nearly my favourites.

One mark each :—Ellie, Le Cerf Agile, Poodle, A. G. S. McCulloch, Ossian, The Eda, Socrates, Toby, Princess Ida, Skye, Edith, Mikado, Scrooge.

**Notices to Correspondents.**

In future I shall give one, two, or three marks for the letters, according to their merit, and I trust all will try to gain the highest score.

LE CERF AGILE.—It will make no difference. Thank you for puzzle.

EDITH.—All letters must be addressed *Puzzle Editor*.

### Amusements with Profit.

PRIZE COMPETITIONS.

**Quarterly Prize Competition.**
Begins 23rd July ; ends 15th October.
**A Prize of Three Guineas**
will be awarded to the Competitor who shall have sent in the largest number of correct or best answers. No one will be permitted to win the Three Guinea Prize twice in any one year, but we shall award annually an additional
**Prize of Five Guineas**
to the competitor who shall have sent in the largest number of correct or best answers during the twelve months.

**No. 1. Double Acrostic.**

A noise that is often prelude to a storm ;
In this Corsican town a great leader was born ;
A concourse soon gathered when disputes arise ;
One who prophetical wonders descries ;
On nights dark and clear how brightly I gleam ;
A tormenting insect whose delight is to sting ;
A warlike goddess, reversed read her name ;
Cut the tail of a shell-fish, no process of pain ;
He who aids the idea in its efforts to shoot ;
The first garden in which flowers ever took root ;
A king of old Rome of selfish renown.

Now the initials and finals kindly read down,
When the former will bring a known poem to view.
And the author be shown by the latter to you.

**No. 2. Enigma.**

No bird that ever flits the sky
Can boast such varied plumes as I ;
And yet I ne'er was known to fly,
Except my wings were broken.

**Answers.**

No. 23. DOUBLE ACROSTIC.

C olinderie S
H ygei A
U sefu L
R ossett I
C hao S
H ore B
I mprompt U
L aboure R
L apidar Y

Correct answers have been received from Ellice, Common Sense, Dolphin, Aralc, Tabs, K. M., Gretta, Letheàdor, Dawnlover, Mater, Mrs. Bedingfeld, Esrun.

No. 24. ENIGMA.

Cap - rice = Caprice.

Correct answers have been received from Fred C., Mrs. Bedingfeld, Canice, Ellice, Common Sense, Aralc, Tabs, K. M., Gretta, Letheàdor, Dawnlover, Mater, Esrun.

### THE IN- AND OUT-PATIENTS' HOSPITAL DIARY.

This Diary is so arranged as to make some portion of it appear every week, and the whole in each monthly part. It has been very difficult to compile, and corrections will at all times be gratefully received. It has been suggested that similar information about the larger Provincial and Scotch Hospitals would be useful to many readers. We should be glad to hear if this is felt to be the case.

### CHILDREN'S DISEASES.—*continued.*

| Hospitals and Terms of Admission. | Physicians, Surgeons, and Medical Officers, with Days and Hours of Attendance. |
|---|---|
| Samaritan Free Hospital for Women and Children, Lower Seymour Street, W.; Branch, 1, Dorset St., W. Sec., Mr. G. Scudamore. In-patients must be between 2 and 12. Out-patients Department (free) is at Edward's Mews, Duke Street, W. | *In-Physician*, Dr. W. H. Day. Daily, 2<br>*In-Surgeon*, Mr. W. A. Meredith. Daily, 2<br>*Out-Physicians*, Drs. M. Prickett and Amand Routh, W. S. 1.30<br>*Out-Surgeons*, Messrs. W. A. Meredith and J. D. Malcolm, M. Th.; A. H. G. Doran and W. S. A. Griffith, Tu. F., 1.30 |
| Victoria Hosp. for Children, Queen's Road, Chelsea, S.W. Sec., Capt. W. C. Blount, R.N. Age of boys, 2 to 12 years; age of girls, 2 to 16. Admission for in-patients by subscriber's letter. Accidents and urgent cases free at all times | *In-Physicians*, Drs. J. Evans, M. Th. 2; Ridge-Jones, Tu. F. 2<br>*In-Surgeons*, Messrs. Pick, Tu. F. 9.15; Clutton, M. Th. 4.30<br>*Out-Physicians*, Drs. A. Venn, W. S. 9.30; C. Fox, M. Th. 1.30; F. D. Drewitt, Tu. F. 9; J. Philpot, M. Th. 9<br>*Out-Surgeons*, Messrs. F. Churchill, Tu. F. 10; W. Pye, M. Th. 9.30 |
| West London Hospital, Hammersmith rd, W. | Daily, 2. *See* General Hospitals |

### CONSUMPTION AND DISEASES OF THE CHEST.

| Hospitals and Terms of Admission. | Physicians, Surgeons, and Medical Officers, with Days and Hours of Attendance. |
|---|---|
| City of London Hospital for Diseases of the Chest, Victoria Park, E. Sec., Mr. T. Storrar Smith. By subscriber's letter available for 6 weeks. | *In-Physicians*, Drs. J. Thorowgood, S.; E. Smith, M.; J. Fothergill, W.; S. West, F.; G. A. Heron, W.; V. D. Harris, Tu. Hour, 2<br>*Out-Physicians*, Drs. V. D. Harris, Tu.; J. A. Ormerod, Th.; E. C. Beale, Tu. F.; B. Fenwick, W. S.; A. Money, W. S.; L. E. Shaw, M. Th. Hour, 2 |
| Hospital for Consumption, Fulham road, Brompton, S.W. Sec., Mr. H. Dobbin. Admission by letter of recommendation, but, where unobtainable, application may be made to the Secretary at the Hospital. | *In-Physicians*, Drs. E. S. Thompson, M. Th.; C. T. Williams, M. Th.; R. D. Powell, Tu. F.; J. Tatham, W. S.; R. E. Thompson, W. S.; F. T. Roberts, Tu. F. Hours, 2 to 4<br>*In-Surgeon*, Mr. R. J. Godlee, F.<br>*Out-Physicians*, Drs. T. H. Green, J. M. Bruce, M. Th.; J. K. Fowler, W. S.; P. Kidd and C. Y. Biss, Tu. F.; T. D. Acland, W. S. Hour, 12 |
| North London Hospital for Consumption and Diseases of the Chest, Mount Vernon, Hampstead, N. Sec., Mr. L. F. Hill. Admission by subscriber's letter available for 6 weeks. | *In-Physicians*, Drs. A. Evershed, Tu.; B. O'Connor, F.; D. Pidcock, F. Hour, 10.30<br>*Out-Physicians*, Drs. B. O'Connor, D. Pidcock, J. E. Squire. Daily, 2 (attend at Out-patients' Branch, 216, Tottenham Court Road, W.) |
| Royal Hospital for Diseases of the Chest, 231, City Rd., E.C. Sec., Mr. John J. Austin. By Governor's letter, available for six weeks | *In-Physicians*, Drs. Hensley, W.; Gilbart-Smith, M.; Finlay, Th.<br>*In-Surgeon*, Mr. A. Pearce-Gould, M. Tu. W. Th. F. 2, S. 9<br>*Out-Physicians*, Drs. White, Tu. F.; Pringle, M. Th.; O. Browne, W. S.; A. Davies, M. F.; H. Habershon, W. S.; E. Stewart, Tu. Th. Hour, 1; S. 9 |
| Royal National Hosp. for Consumption and Diseases of the Chest, Ventnor. Offices, 34, Craven St., Strand, W.C. Sec., Mr. E. Morgan. Admits only incipient cases by Governor's letter. | *In-and Out-Physicians*, Drs. J. G. Sinclair Coghill, and R. Robertson attend daily (at Ventnor) on out-patients at 11; on in-patients at noon<br>*In- and Out-Surgeons*, Messrs. Whitehead, Williamson |

### EAR CASES.

| Hospitals and Terms of Admission. | Physicians, Surgeons, and Medical Officers, with Days and Hours of Attendance. |
|---|---|
| Central London Throat and Ear Hosp., Gray's Inn Rd., W.C. Sec., Mr. R. Kershaw. Free, but when able, a small payment is expected | *In- and Out-Surgeons*, Mr. Lennox Browne, M, Th. 2.30; Dr. Orwin, W. S. 2.30; Dr. Grant, Tu. 5, Th. 2.30; Messrs. P. S. Jakins, M. W. 2.30; F. 5; T. Carmalt Jones, M. Th. S. 2.30 |
| Charing Cross Hospital, West Strand, W.C. | *In- and Out-Surgeon*, Mr. Cantlie, F. 9 |
| Great Northern Central Hospital, Caledonian Rd, N. | *In- and Out-Surgeon*, Mr. A. E. Cumberbatch, W. 9 |
| Guy's Hospital, St. Thomas's Street, S.E. | *In- and Out-Surgeon*, Mr. Purves, Tu. F. 12.30 |
| Hospital for Diseases of the Throat, Golden Square, W. Sec., Mr. W. T. Sharp | *Physicians*, Drs. Wolfenden, Tu. F. 2.30; Macdonald, Tu F. 6.30; Bond, W. S. 2.30<br>*Surgeon*, Mr. T. M. Hovell, M. Th. 2.30 |

### EAR CASES—*Continued.*

| Hospitals and Terms of Admission. | Physicians, Surgeons, and Medical Officers, with Days and Hours of Attendance. |
|---|---|
| King's College Hospital, Portugal Street, W.C. | *In- and Out- Surgeon*, Mr. Pritchard Th. 2.30 |
| London Hospital, Whitechapel Road, E. | *In- and Out-Surgeons*, Dr. Woakes, Mr. T. M. Hovell, S. 9 |
| Middlesex Hospital, Berners Street, W. Free. | *Out-Surgeon*, Mr. Hensman, Tu. 9 |
| National Hosp. for the Paralysed, etc., Queen Sq., W.C. | *In- and Out-Surgeon*, Mr. A. E. Cumberbatch, W. 2 |
| North West London Hospital, 18, Kentish Town Rd. | *Out-Surgeon*, Mr. Collier, M. 1.30 |
| St. Bartholomew's Hospital, Smithfield, E.C. | *Surgeon*, Mr. Cumberbatch, Tu. F. 2 |
| St. George's Hospital, Hyde Park Corner, S.W. | *In- and Out-Surgeon*, Sir W. B. Dalby, Tu. 1.30 |
| St. Mary's Hospital, Cambridge Place, Paddington, W. | *In- and Out-Surgeon*, Mr. G. P. Field, M. Thur. 3 |
| St. Thomas's Hospital, Westminster Bridge Road, S.E. | *Out-Surgeon*, Mr. Clutton, M. 1.30 |
| University College Hospital, Gower Street, W.C. | *In- and Out-Surgeon*, Mr. Barker, Th. 9 |
| Westminster Hospital, Broad Sanctuary, S.W. | *In- and Out-Surgeon*, Mr. Barrow, Tu. F. 9 |

### EYE DISEASES.

| Hospitals and Terms of Admission. | Physicians, Surgeons, and Medical Officers, with Days and Hours of Attendance. |
|---|---|
| Central London Ophthalmic Hosp., 238a, Gray's Inn Rd., W.C. Sec., Mr. G. H. Leah. Admission free | *Surgeons*, Messrs. T. Archer, G. Abbott, W. Charnley, W. Jesson, E. Clarke, J. R. Kemp and Granville Barker (House Surgeon). Daily, 1 |
| Evelina Hosp. for Sick Children, Southwark Bridge Rd. | *Surgeon*, Dr. W. Brailey, Th. 1 |
| Great Northern Central Hospital, Caledonian Rd., N. | *Surgeon*, Mr. Hutchinson, Jnr., Tu. F. 9.30 |
| Guy's Hospital, St. Thomas's Street, S.E. | *Surgeons*, Mr. Higgens, Dr. Brailey Tu. F. 12.30 |
| Hospital for Sick Children, 49, Gt. Ormond Street, W.C. | *Surgeon*, Mr. R. Marcus Gunn, Tu. 2 |
| King's College Hospital, Portugal Street, W.C. | *Surgeon*, Mr. McHardy, M. W. Th. 1.30 |
| London Hospital, Whitechapel Road, E. | *Surgeons*, Messrs. Waren Tay, S. 9; Eve, Tu. 9 |
| Middlesex Hospital, Berners Street, W. | *Surgeon*, Mr. Lang, Tu. F. 9 |
| National Hosp. for the Paralysed, etc. Queen Sq., W.C. | *Surgeons*, Messrs. R. Brudenell Carter F. 4; R. Marcus Gunn, Tu. 4 |
| North-West London Hosp. 18, Kentish Town Rd., N.W. | *Surgeon*, Dr. W. Collins, F. 3.30 |
| Paddington Green Children's Hosp., Paddington Grn. | *Surgeon*, Mr. W. H. H. Jessop, Th. 9 |
| Royal Free Hospital, Gray's Inn Road, W.C. | *Surgeon*, Mr. Mackinlay, M. F. 9 |
| Royal London Ophthalmic Hospital, Blomfield Street, Moorfields, E.C. Sec., Mr. R. J. Newstead. Admission free to the poor. | *Surgeons*, Messrs. J. W. Hulke, W. S.; G. Lawson, Tu. F.; J. Couper, Tu. F.; W. Tay, M. Th.; J. Tweedy, Tu. F.; E. Nettleship, W. S.; R. M. Gunn, W. S.; Lang, M. Th. Hours, 8 to 10 |
| Royal South London Ophthalmic Hosp., 6, St. George's Circus, S.E. Sec., Mr. C. Comyn. Free to the necessitous poor; or by payment | *Surgeons*, Messrs. Watson, McHardy. Daily, 2 |
| Royal Westminster Ophthalmic Hospital, King William Street, W.C. Sec., Mr. T. Beattie-Campbell. Poor and accidents free | *In- and Out-Surgeons*, Messrs. Power, M. F.; Frost, M. Th.; Rouse, Tu. S.; Hartridge, Tu. F.; Macnamara, M. Th.; Cowell, W. S.; Juler, W. S. Hour, 1 |
| St. Bartholomew's Hospital, Smithfield, E.C. | *Surgeons*, Messrs. Power, Th. 2; Vernon, Tu. S. 2.30 |
| St. George's Hospital, Hyde Park Corner, S.W. | *Surgeons*, Messrs. B. Carter, Frost, M. Th. 2; F. 1.15; W. S. 2 |
| St. Mary's Hospital, Cambridge Place, Paddington. W. | *Surgeons*, Messrs. A. Critchett, H. Juler, Tu. F. S. 9 |
| St. Thomas's Hospital, Westminster Bridge Road, S.E. | *Surgeons*, Messrs. Nettleship, Tu. Th. F. 1.30; Lawford, M. W. 1.30 |
| University College Hospital, Gower Street, W.C. | *Surgeon*, Mr. Tweedy, M. Tu. Th. F. 2 |
| West London Hospital, Hammersmith Road, W. | *Surgeons*, Messrs. B. Vernon, H. Dunn, Tu. Th. S. 2 |
| Westminster Hospital, Broad Sanctuary, S.W. | *Surgeon*, Mr. Cowell, M. Th. 2 |
| Western Ophthalmic Hosp. 153 and 155, Marylebone Rd., W. Sec., Mr. G. E. Manton. By letter or small payments; free to poor | *Surgeons*, Drs. Miller, M.; Collins, F.; Wheatly, M. Th.; Messrs. Carmal, Jones, W.; Buxton, Tu. F.; Nunn, W. S. Hour, 2 |

### The In- and Out-Patients' Hospital Diary.

### ORTHOPÆDIC CASES.

| Hospitals and Terms of Admission. | Physicians, Surgeons, and Medical Officers, with Days and Hours of Attendance. |
|---|---|
| City Orthopædic Hospital, 27, Hatton Garden, E.C. Sec., Mr. E. Dereuth. Admission free | *Surgeons*, Mr. E. J. Chance and House-Surgeon. M. Tu. Th. F. 2.30. New cases Tu. F. 2.30 |
| National Orthopædic Hospital for the Deformed, 234, Gt. Portland St., W. Sec., Mr. H. Canning. Free to poor; others by letter or payment | *In-Physician*, Dr. C. Beevor. *In- and Out-Surgeons*, Messrs. F. R. Fisher, M. Th. 2; W. J. Roeckel, Tu. F. 2; E. M. Little, W. 1 |
| Royal Orthopædic Hospital, 297, Oxford Street, W. Sec., Mr. B. Maskell. By Governor's letter | *Surgeons*, Messrs. B. E. Brodhurst, H. A. Reeves, C. Read. W. E. Balkwill, H. F. Baker. Daily, 1 |
| St. Bartholomew's Hospital, Smithfield, E.C. | *Surgeon*, Mr. Walsham, M. 2.30 |
| St. George's Hospital, Hyde Park Corner, S.W. | W. 2. *See* General Hospitals |

### PARALYSIS, EPILEPSY, AND DISEASES OF THE NERVOUS SYSTEM GENERALLY.

| | |
|---|---|
| Hospital for Epilepsy and Paralysis, and other Diseases of the Nervous System, 32, Portland Terrace, Regent's Park Road, N.W. Sec., Mr. Howgrave Graham. By contributor's letter, or payment according to means, but free to necessitous poor | *In-Physicians*, Drs. Hughes Bennett, M.; G. Ogilvie, Tu.; J. Althaus, Th. Hour, 2. *Out-Physicians*, Drs. H. Bennett, M.; G. Ogilvie, Tu.; W. H. Syers, W.; J. Althaus, Th.; Laycock, F. Hour, 2. *In- and Out-Surgeons*, Messrs. J. Bloxam, A. P. Gould, when required |
| National Hospital for the Paralysed and Epileptic, Queen Square, Bloomsbury, W.C. Sec. and Director, Mr. B. B. Rawlings. Most of the beds are reserved free to the necessitous poor; the others are for patients who contribute a Guinea a week | *In-Physicians*, Drs. C. B. Radcliffe, M.; J. S. Ramskill, Tu.; T. Buzzard, W.; J. H. Jackson, F. Hour, 2.30. *In-Surgeons*, Messrs. W. Adams, M. 3; V. Horsley, F. 2.30. *Out-Physicians*, Drs. W. R. Gowers, M.; H. C. Bastian, Tu.; J. A. Ormerod, Tu. W.; T. Buzzard, W.; D. Ferrier, F.; C. E. Beevor, M. F. Hour, 2.30 |
| National Hospital for Diseases of the Heart and Paralysis, 32, Soho Sq., W. Sec., Capt. F. Handley. In-patients require recommendation of Life Governor. | *In-Physicians*, Drs. A. Duncan, J. Murray, Stretch Dowse, M. W. Th. *In-Surgeon*, Mr. Graham Bennett, Th. *Out-Physicians*, Drs. R. Verley, J. Murray, Stretch Dowse, Tu. Th. F. 3; M. W. 3 and 8. *Out-Surgeon*, Mr. G. Bennett, Th. 3 |
| West End Hospital for Diseases of the Nervous System, 73, Welbeck St., W. Hon. Sec., Mr. H A. Dowell. Children only as in-patients. Out-patients by letter or payment. | *In- and Out-Physicians*, Drs. H. Tibbits, M. Th. 1.30; Stretch-Dowse, Tu. W. 1.30; S. H. Armitage, W. Tu. F. 6. *In- and Out-Surgeon*, Mr. Benson |

### DISEASES OF THE SKIN.

| | |
|---|---|
| Charing Cross Hospital, West Strand, W.C. | *Physician*, Dr. Sangster, M. 2 |
| Guy's Hospital, St. Thomas Street, S.E. | *Physicians*, Drs. Hale-White, Pye-Smith, Tu. 12 |
| Hospital for Diseases of the Skin, 52, Stamford St., S.E. Sec., Mr. S. Hayman. Free to the necessitous; also by payment | *Physician*, Dr. F. J. Payne. *Surgeons*, Messrs. J. Hutchinson, Waren Tay, and Wyndham Cottle. (Out-patients seen M. W. 3, Tu. Th. F. 2) |
| King's College Hospital, Portugal Street, W.C. | *Physician*, Dr. Duffin, F. 1 |
| London Hospital, Whitechapel Road, E. | *Physician*, Dr. S. Mackenzie, Th. 9 |
| Middlesex Hospital, Berners Street, W. | *Physician*, Dr. R. Liveing, Tu. 4 |
| North-West London Hosp. 18, Kentish Town Rd., N.W. | *Physician*, Dr. J. Stowers, Tu. 1.30 |
| Paddington Green Children's Hospital, Paddington | *Physician*, Dr. T. Colcott Fox, S. 9.15 |
| St Bartholomew's Hospital, Smithfield, E.C. | *Surgeon*, Mr. H. Cripps, F. 1.30 |

### DISEASES OF THE SKIN.—*Continued.*

| Hospitals and Terms of Admission. | Physicians, Surgeons, and Medical Officers, with Days and Hours of Attendance. |
|---|---|
| St. George's Hospital. Hyde Park Corner, S.W. | *Physician*, Dr. Cavafy, W. 1.30 |
| St. John's Hospital for Diseases of the Skin, 45, Leicester Sq., W.C. Branch, Markham Square, Chelsea, S.W. Sec., Mr. St. Vincent Mercier. In-patients by letter; out-patients free or by small payment | *Physicians*, Drs. Bourns, Campbell, Dow. Harries, Robinson. Daily 2 and 7. Markham Sq. Branch, M. 10 and Th. 10 and 7. *Surgeon*, Mr. J. Milton, Tu. |
| St. Mary's Hospital, Cambridge Place, Paddington, W. | *Surgeon*, Mr. Morris, M. Th. 9.30 |
| St. Thomas's Hospital, Westminster Bridge Road, S.E. | *Physician*, Dr. Payne, W. 1.30 |
| University College Hospital, Gower Street, W.C. | *Physician*, Dr. Crocker, W. 1.30, S. 9.15 |
| Westminster Hospital, Broad Sanctuary, S.W. | *Physician*, Dr. T. C. Fox, W. 1.30 |

### STONE AND OTHER DISEASES OF URINARY ORGANS.

| | |
|---|---|
| London Hospital, Whitechapel Road, E. | Daily, 1 30. *See* General Hospitals |
| St. Peter's Hospital for Stone and Urinary Diseases, Henrietta Street, W.C. Sec., Mr. W. E. Scott. Admission free. Only women and children are seen on Fridays. Operation day, Wednesday | *In-Surgeons*, Messrs. W. J. Coulson, F. R. Heycock. *Out-Surgeons*, Messrs. E. H. Fenwick, M. 2, S. 4 to 7; F. S. Edwards, M. 5 to 7, Th. 2; Heycock, Tu. 2, W. 5 to 7, F. 2 |

### DISEASES AND IRREGULARITIES OF THE TEETH.

| | |
|---|---|
| Belgrave Hosp. for Children, 77 & 79 Gloucester St., Pimlico | *Surgeon*, Mr. G. W. Payne, W. 9 |
| Charing Cross Hospital, West Strand, W C. | *Surgeon*, Mr. Fairbank, M. W. F. 9.0 |
| Dental Hospital of London, Leicester Sq., W.C. Sec., Mr. J. F. Pink. Poor free. For special operation a Governor's letter is required | *Surgeons*, Messrs. S. Bennett, Canton, Gregson, Hepburn, Hern, Matheson, Parkinson, Read, Rogers, Truman, Underwood, Woodhouse. Daily, 9 to 11 |
| Evelina Hosp. for Sick Children, Southwark Brdg. Rd. | *Surgeon*, Mr. R. Denison Pedley, Tu. 9 |
| German Hospital, Dalston lane, Dalston, E. | *Surgeons*, Messrs. A. P. Reboul, C West, Th. 10 to 11 |
| Great Northern Central Hospital, Caledonian Rd, N. | *Surgeon*, Mr. E. Keen, W. 1.30 |
| Guy's Hospital, St. Thomas Street, S.E. | *Surgeons*, Messrs. Moon, Pedley, Th. F. 12.30 |
| Hospital for Consumption, Brompton, S.W. | *Surgeon*, Mr. C. J. Noble, W., when wanted |
| Hospital for Sick Children, Gt. Ormond Street, W.C. | *Surgeon*, Mr. Cartwright, W. 9 |
| King's College Hospital, Portugal Street, W.C. | *Surgeon*, Mr. Cartwright, Tu. F. 10 |
| London Hospital, Whitechapel Road, E. | *Surgeon*, Mr. A. W. Barrett, Tu. 9 |
| London Temperance Hosp., Hampstead Road, N.W. | *Surgeon*, Mr. Adolphus Alexander, M. 2 |
| Metropolitan Free Hospital, Gray's Inn Road, W.C. | *Surgeon*, Mr. G. Curnock, Tu. Th. S. 9 |
| Middlesex Hospital, Berners Street, W. | *Surgeons*, Messrs. Bennett, M. 9.30; W. 9; Hern, W. 9, F. 9.30 |
| National Dental Hospital, 149, Gt. Portland Street, W. Sec., Mr. A. G. Klugn. The poor, and all urgent cases admitted free; others by Subscriber's letter | *Surgeons*, Messrs. H. Weiss, W. Weiss, M.; A. Smith, G. Bradshaw, Tu.; G. A. Williams, M. Davis, W.; A. F. Canton, H. G. Read, Th.; T. Gaddes, W. S. Thomson, F.; H. Rose, W. R. Humby, S. Hours 9 to 11 |
| National Orthopædic Hospital, 234, Gt. Portland st. W. | *Surgeon*, Mr. H. Harris |

